# Neuropsychiatric Symptoms (NPS) in patients with pure Vascular Dementia (VaD) and Mixed Dementia (MD) from a memory outpatient clinic in southeast Brazil

**DOI:** 10.1590/S1980-57642013DN70300006

**Published:** 2013

**Authors:** José Ibiapina Siqueira-Neto, Octávio Marques Pontes-Neto, Francisco de Assis Carvalho do Vale, Júnia Vieira dos Santos, Paulo Marcelo Gondim Sales, Júnia Vieira dos Santos, Antônio Carlos Santos

**Affiliations:** 1 MD, PhD. Associate Professor of Neurology, Clinical Medicine Department, Faculty of Medicine, Federal University of Ceará, Brazil.; 2 MD, PhD. Associate Professor of Neurology, Department of Neuroscience and Behavior Sciences, University of São Paulo, University Hospital of Ribeirao Preto, Brazil.; 3 MD, PhD. Adjunct Professor of Neurology, Federal University of São Carlos, Brazil.; 4 MD, PhD. Medical Doctor Candidate, Federal University of Ceará, Faculty of Medicine, Brazil.; 5 MD, PhD. Associate Professor of Neuroradiology in Center of Sciences of Imaging and Medical Physics of São Paulo University-Ribeirão Preto, Ribeirão Preto University Central Hospital, Brazil.

**Keywords:** vascular dementia, neuropsychiatric symptoms, vascular risk factors, developing countries

## Abstract

Vascular Dementia (VaD) and Vascular Cognitive Impairment (VCI) are increasingly common worldwide. Nevertheless, the clinical-neuropsychiatric profile of these patients at presentation is still poorly characterized in developing countries.

**OBJECTIVE:**

We aimed to characterize the prevalence of neuropsychiatric symptoms, as well as the clinical and cognitive profile of patients with VaD and VCI in our tertiary University outpatient cognitive clinic.

**METHODS:**

We reviewed data on 253 patients diagnosed with VaD or VCI at our center between January 1996 and December 2005, located in an industrial region of the state of Sao Paulo, southeast Brazil. We excluded 19 patients who did not complete the medical investigation or who did not meet the clinical or neuroimaging criteria for vascular dementia. We collected socio-demographic data, educational level, vascular risk factors, behavioral and neuropsychological symptoms and cognitive complaints at presentation.

**RESULTS:**

Two hundred and thirty-four cases were included in this analysis. The mean age was 67.77±10.35 years; 72% were males and 82% had less than four years of education (average 2.84±2.96 years). The initial Clinical Dementia Rating score was 2 & 3 in 68%. A total of 185 patients had neuropsychiatric symptoms distributed in main categories as follows: psychosis (52.6%), hallucinations (23.5%), psychomotor agitation (22.5%), depression (17.5%) and apathy (17.5%). Hypertension and previous stroke were the most prevalent risk factors.

**CONCLUSION:**

We found a high prevalence of neuropsychiatric symptoms. The clinical-neuropsychiatric profile of patients presenting to cognitive clinics in developing countries may differ greatly to that of more developed nations. These characteristics may have implications for public health strategies.

## Abstract (pt)

Demência Vascular (DV) e comprometimento cognitivo vascular (CCV) são diagnósticos cada vez mais relatados em todos os continentes. Entretanto, o perfil dos sintomas comportamentais e psicológicos das demências (SCPD) nos pacientes com DV é ainda pouco descrito e caracterizado, nos países em desenvolvimento.

**OBJETIVO:**

Determinar a prevalência dos SCPD, o perfil de manifestações neuropsiquiátricas e cognitivas, nos pacientes com DV no ambulatório de Neurologia Cognitiva e Comportamental (ANCC), do Hospital das Clínicas da Universidade de São Paulo em Ribeirão Preto (HCRP-FMUSP).

**MÉTODOS:**

Revisamos os prontuários de 253 pacientes diagnosticados com DV ou CCV atendidos entre janeiro de 1996 e dezembro de 2005. Excluímos 19 pacientes que não completaram a investigação diagnóstica, não preencheram os critérios clínicos do DSM-IV, e neuroimagem não disponível para análise. Coletamos dados sócio-demográficos, nível de escolaridade, fatores de risco vascular, e SCPD da consulta de admissão.

**RESULTADOS:**

234 pacientes foram incluídos nesta análise. A idade média global foi de 67,77±10,35; com 38% de mulheres; escolaridade de 2,84±2,96 anos; MEEM inicial 13,22±7,00, e 68% de CDR 2 & 3. 79% de pacientes com SCPD foram divididos nas seguintes categorias: psicose (52,6%), alucinações (23,5%), agitação (22,2%), depressão (17,5%) e apatia (17,5%) foram às manifestações mais prevalentes. Hipertensão Arterial e AVC prévio foram os fatores de risco de maior prevalência.

**CONCLUSÃO:**

Verificou-se elevada prevalência de SCPD em DV. O perfil destas alterações neuropsiquiátricas na DV mostrou uma tendência na direção de sintomatologia psicótica, devendo ser objeto de mais pesquisas, pois os padrões em nações desenvolvidas podem ser substancialmente diferentes.

## INTRODUCTION

Vascular Dementia (VaD) is increasingly common in cognitive clinics worldwide. This type of Dementia is probably the second-most-common cause of dementia after Alzheimer's disease.^1-5^ Prevalence rates of post-stroke dementia (PSD), one of the most frequent subtypes of VaD, range from 12.2% to 31.8% in the first year after stroke^1^ and VaD is estimated at between 20% and 40%.^2-5^ Mixed dementia seems to be more prevalent than the pure form of VaD.^2-5^ Some community and hospital-based studies in Brazil have suggested a prevalence ranging from 9.3% to 24.9% for VaD.^6-8^ In the last few years, the term Vascular Dementia has been partially substituted, now more commonly referred to as VCI (Vascular Cognitive Impairment).^9^ Nevertheless, in this paper we have used VaD because the vast majority of our patients had dementia.

The most used group of criteria for diagnosing VaD includes: ICD-10,^10^ DSM-IV,^11^ NINDS-AIREN,^12^ and ADDTC (State of California Alzheimer's Disease Diagnostic and Treatment Centers).^13^ The NINDS-AIREN (National Institute of Neurological Disorders and Stroke and Association Internationale pour la Recherché et l'Enseignement en Neuroscience)^11^ criteria is used preferentially in controlled assays because it is very specific, but less sensitive. The Hachinski Score^14^ is easily applied, sensitive, but less specific for subcortical dementia and does not include imaging. At present, a better group of criteria is not available (DSM-IV, ICD-10, ADDTC and NINDS-AIREN).^10-14^

Neuropsychiatric symptoms are mainly classified into mood, psychotic disorders, and frontal manifestations.^3,15-19^ Paranoid delusions were the most common psychotic symptom reported in Alzheimer´s dementia, particularly at late stages.^15^ The prevalence of Behavioral and Psychological Symptoms of Dementia (BPSD) attains 90% during the course of illness in subjects with Alzheimer's disease, but there are scant references to this profile in VaD for Brazilian patients. With the exception of Psychosis and Depression in AD, there are few consensus diagnostic criteria for BPSD in dementia.^20-23^

The aim of this study was to determine the prevalence of BPSD at the 1^st^ consultation and characterize the profile of BPSD of patients with VaD and VCI at our public tertiary university outpatient cognitive clinic located in Ribeirao Preto city, Sao Paulo, Brazil.

## METHODS

**Subjects.** This was a retrospective study involving two-hundred fifty-three consecutive patients with a diagnosis of either Vascular Dementia or VCI followed at the Memory Clinic at the University Hospital, Ribeirao Preto - Southeast - Brazil. This is a regional tertiary clinic which is part of the Sao Paulo Health Care System. A thorough review of all medical records of the patients seen at this service from January 1996 to December 2005 was carried out. This study was approved by our Institutional review board.

**Diagnosis of vascular dementia.** Two hundred thirty-four patients with a diagnosis of vascular dementia according to the Diagnostic and Statistical Manual IV (American Psychiatric Association, 1994)^11^ criteria were included. Patients who did not complete the medical investigation or who did not meet the clinical or neuroimaging criteria for vascular dementia were excluded (N=19).

**Medical investigation.** The dementia work-up included: complete medical history, sociodemographic variables, risk factors, clinical examination, neurological examination, screening questionnaires and scales for cognitive disorders and brain MRI or CT-Scan. Other neuroimaging studies, such as SPECT, MRI angiography, carotid duplex scan, and conventional angiography, completed the investigation in selected cases. Routine laboratory tests for dementia were performed, which included: complete blood count, renal and liver function tests, glucose, cholesterol total and fractions, calcium and phosphate levels, serum vitamin B12, serum folic acid, thyroid function tests, HIV serology and VDRL, and transthoracic echocardiogram. Other blood tests for Chagas disease, thrombophilic state, auto-immune diseases, hematologic diseases and cardiac tests (holter, transesophageal echocardiogram and coronarography) were also performed electively.

The cognitive screening battery included the Mini-Mental State Examination (MMSE), and CDR (Clinical Dementia Rating) was performed in all cases. The Neuropsychiatric Inventory (NPI), HIS (Hachinski Ischemic Score), Pfeffer Functional Index, Geriatric Depression Scale were not performed routinely during the time of the study. The NINDS-AIREN and ADDTC criteria for VD were also not applied. Formal complete neuropsychological evaluation was carried out in only a few cases, precluding consistent analysis.

The majority of the patients were studied with Brain MRI (1.5 Tesla). After collection of all clinical and neuroimaging data we discussed the findings in a consensus discussion with the members of our team, including 03 cognitive neurologists and an experienced neuroradiologist for classification ascertainment. The following parameters were evaluated on imaging: [1] White matter changes, [2] Multiple cortical infarcts, [3] Subcortical Infarcts, [4] Strategically located Infarcts, [5] Hemorrhagic Lesions, [6] Ventricular dilatation, [7] Cerebral Atrophy, [8] Symmetric Temporal Atrophy (not proportional to age). Fazekas grade was applied to measure the burden of white matter changes as a cut-off point to evaluate subcortical lesions.

**Neuropsychiatric symptoms.** The classification of psychiatric symptoms was obtained from chart review based on the structured admission interview questionnaire of our memory clinic. The protocol includes questions about all major neuropsychiatric domains. The symptoms of psychotic and mood behavior were also registered.

**Statistical analysis.** The analyses were undertaken with the aid of the Statistical Package for the Social Sciences software package (SPSS). The data were described through frequency distributions of categorical variables, mean values and standard (mean±SD) deviations for continuous variables with a normal distribution. Continuous data were compared using the Chi-square test (p<0.05) when normally distributed or with the Mann-Whitney U-test if non-normally distributed (univariate analysis), while categorical variables were compared by cross-tabulation.

## RESULTS

**Demographic and clinical variables.** The study population exhibited a Male : Female ratio of 1:0.6. All two hundred thirty-four patients had their educational profile documented. A total of 34.62% of subjects were illiterate and only 4.7% had university level education or higher. Socio-demographic variables are given in Table 1.

**Table 1:** Socio-demographic variables (N=234)

Age (mean±SD)	Global	67.77±10.35
	Male	66.97±10.18
	Female	69.09±10.56
Female gender (%)		38.03
Education	Years (Mean±SD)	2.84±2.96

**Figure 1:**
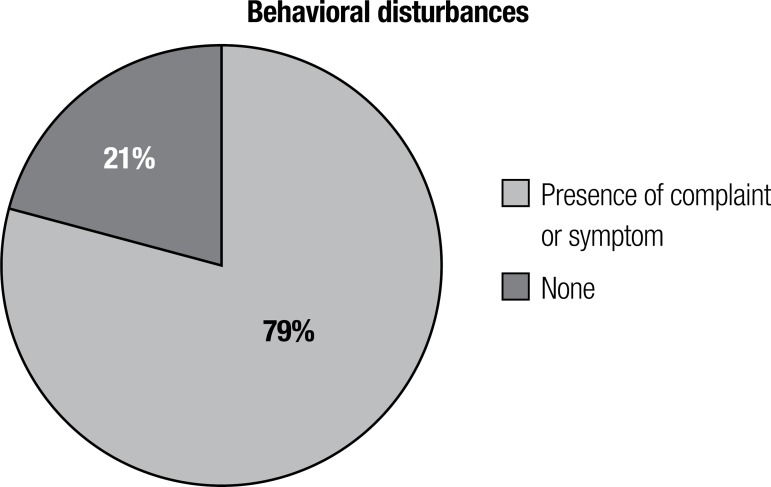
NPS complaints at first consultation.

**Non-cognitive complaints at presentation.** The prevalence of non-cognitive complaints at the admission consultation is shown in Figure 1.

**Major clinical findings.** At the first inquiry, the mean time elapsed from the first complaint to clinical attendance at our memory clinic was 2.69±2.50 years (range 0-20). Two hundred and twenty-nine individuals met the DSM IV criteria for dementia while only five patients (2.14%) presented vascular cognitive impairment (VCI). Our series had a greater prevalence of isolated VaD, with 123 cases, versus 111 cases of mixed dementia. Other clinical findings are described in Table 2.

**Table 2:** Major clinical findings observed at first interview.

Initial MMSE (Mean±SD)	13.22±7.00
CDR 0.5&1 (%)	32.05
CDR 2 & 3 (%)	67.95
Delay in attendance^1^ (Mean±SD)	2.69±2.50
DSM-IV criteria for VD (%)	97.86
Vascular cognitive impairment (%)	2.14
Previous stroke (%)	23.93
Psychotropic drug Use (%)	79.91
Cholinesterase inhibitor use (%)	19.66

CDR: Clinical Dementia Rating; SD: Standard Deviation; MMSE: Mini-Mental State Examination; VD: Vascular Dementia.

1 Time elapsed from perception of chief complaint to seeking treatment at our memory clinic, expressed in years.

**Overview of comorbidities and vascular risk factors.** All patients had comorbidities documented, where hypertension was present in 80.80% and previous stroke in 42.70%, with significance difference in gender for the latter condition. This data can be found in Table 3.

**Table 3:** Possible risk factors (%).

Variable	Gender		
	**Males (N=145)**	**Females (N=89)**	**Both (N=234)**
Alcohol abuse*	28.97	1.12	18.38
Tobacco abuse*	26.90	5.62	18.80
Diabetes mellitus	22.07	29.21	24.79
Dyslipidemia	6.90	8.99	7.69
Hypertension	79.31	83.15	80.77
Stroke*	53.79	37.08	47.43
Transient ischemic attack	5.52	5.62	5.56
Atrial fibrillation	6.21	7.87	6.84
Ischemic cardiopathy	10.34	14.61	11.97

* Significant difference observed between groups on chi-square (P<0.05).

**Figure 2:**
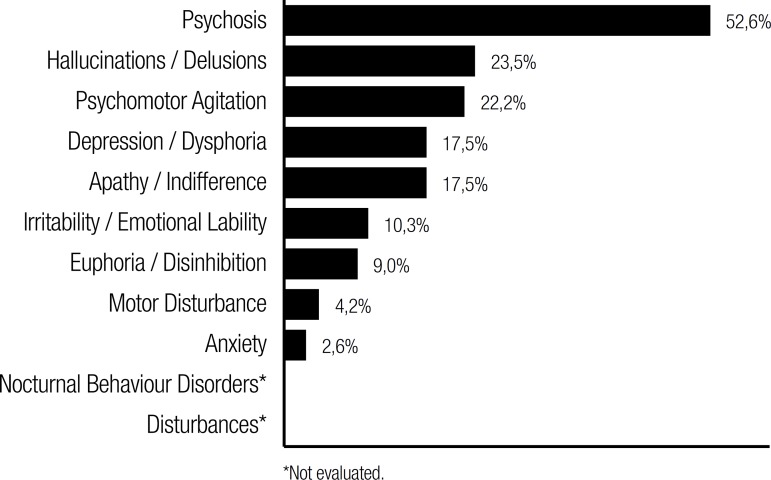
Neuropsychiatric Abnormalities in patients with behavioral complaints or detected symptoms (expressed in percentage).

**Characteristics of behavioral disturbances in dementia patients.** The most common presenting behavioral abnormality was hallucination (23.50%), followed by agitation (22.20%) and depression (17.50%). Figure 2 summarizes the psychiatric abnormalities in patients with behavioral complaints or detected symptoms.

## DISCUSSION

The high prevalence and clinical significance of psychiatric disturbances in dementia are now receiving increased attention. Most of the series available estimate the prevalence of neuropsychiatric signs and symptoms in dementia of the Alzheimer type, and a broad range of rates from 60% to 90% has been reported.^3-5,24-27^ In our series, we observed a prevalence at first consultation of 79%, similar to the upper limit cited. This may indicate that in our clinics, the vascular burden may contribute in the form of presentation of dementia.

Particularly noteworthy is the extremely high prevalence of low educational level in our sample. Most series in developed countries have reported an average of 12 years of study or more.^3,17-19,25^ The review by Kalaria NJ et al.^2^ on vascular dementia in developing countries, showed similar results to those found in our series. The substantial impact of educational level on understanding the importance of preventive measures in this situation probably explains the fact that the majority of patients had GDS 2 and 3 at 1^st^ consultation, because most failed to understand at the time of our study that cognitive decline was a consequence of disease and not of aging. However, it can also suggest that nutrition and lifestyle are important factors for NPS. The low diagnosis of VCI is probably the result of this low education.^1,2,19^

The decision to use DSM-IV as the sole criteria for diagnosing vascular dementia is owing to the period the patients were followed and the nature of the retrospective analysis. The material was retrieved from the hospital database of records for 1996-2005 and data bank imaging, where most of the patients at the time of data collection were either unavailable or had deceased. This time interval was established because it coincides with the initial activities of the memory outpatient clinics of our hospital, and we decided to conduct analysis over a period spanning 10 years. This same reason also explains the absence of application of behavioral scales such as the NPI or Behave-AD. The patients were followed at a general dementia outpatient unit, and in the late nineties this scale was not routinely used. We emphasize that our questionnaire, based on the suggestions of Lyketsos^18^ and Olin^21^ for Psychosis and Depression in AD, was very efficient in characterizing these ancillary pictures.

Another interesting point regarding this material concerns the possible risk factors. We observed the same finding as most papers, confirming hypertension as the most prevalent risk factor with no differences between gender.^1,2,19^ Diabetes mellitus and dyslipidemia was also frequent, with no differences again between sexes. Three major risk factors for vascular dementia with significant differences in gender was previous stroke, tobacco use and alcohol abuse, all of which were more prevalent in males. The correlation of these findings with BPSD was not analyzed here but constitutes an interesting issue for further discussion.

The evidence and description of BPSD in other dementia types is notably scarcer than for AD.^3,15-18, 24-27^ This fact *per se* justifies the present work. Vascular dementia may have a somewhat lesser tendency than AD or dementia with Lewy bodies to cause hallucinations and delusional symptoms, although not all studies have verified this.^3,15-18^ Psychosis was very common in our series with vascular dementia (52.6%), and only one previous study reports a high prevalence of 46% among hospitalized patients^25^. We can hypothesize that this result is a consequence of the majority of our patients suffering from moderate-severe dementia. Nevertheless, we emphasize that schooling level in developing countries contributes substantially to this picture. Selecting a population with VCI and mild dementia remains particularly difficult. Therefore, this sample is still valuable because it represents the reality of our memory clinics throughout the country.

There is a dearth of information on depression in dementias other than AD. Some evidence suggests that depression may actually be more common in vascular dementia than in AD. Depression in vascular dementia has been associated with abnormal neurological signs such as extrapyramidal symptoms and grasp reflexes, possibly implicating frontal-subcortical circuits. Our finding of 17.5% depressive symptoms does not corroborate the results verified in reviews, but we have identified some studies in which this percentage is similar.^3^ We may speculate that differential diagnosis of apathy with this condition was not performed properly at the time, and so this prevalence may be greater. The new criteria for depression in dementia certainly influence actual results.^20-23^

An interesting study of neuropsychiatric symptoms across 4 dementia types [Alzheimer's disease (AD), vascular dementia (VAD), dementia with Lewy bodies (DLB), and Parkinson's disease dementia], and 2 mixed groups (AD/VAD and AD/DLB) in a sample of 2,963 individuals from the National Alzheimer's Coordinating Center Uniform Data Set between September 2005 and June 2008, found a significantly higher prevalence of BPSD in VaD, and more complex findings in mixed type dementias^15^. Psychosis was reported in 41% of patients with Alzheimer's disease, including delusions in 36% and hallucinations in 18%. The incidence of psychosis increased progressively over the first 3 years of observation, after which the incidence seemed to plateau. Psychotic symptoms tended to last for several months but became less prominent after 1 year. We do not have data to compare our results with other types of dementia in our clinics but the prevalence of psychosis is very high when correlated with AD series and the presence of BPSD was similarly high at the 1^st^ consultation.^25-27^

In conclusion, our preliminary findings on the prevalence of BPSD in VaD indicate psychosis as the leading picture. Depression is also found at a lower proportion. Low educational level and male gender were also observed. A high prevalence of BPSD was observed at 1^st^ consultation. Possible risk factors found included hypertension and previous stroke. This initial study should be furthered with possible new correlations. We emphasize the importance of documentation concerning neuroimaging in our series toward helping to correctly evaluate vascular burden.
